# Dysmenorrhea pattern in adolescences informing adult endometriosis

**DOI:** 10.1186/s12889-024-17825-2

**Published:** 2024-02-05

**Authors:** Yu Dai, Huangjin Luo, Litong Zhu, Weichun Yang, Haishan Xiang, Qiuling Shi, Ping Jin

**Affiliations:** 1 Department of Gynecology, Shenzhen Maternity and Child Healthcare Hospital, Shenzhen, Guangdong People’s Republic of China; 2 Department of Gynecology, Shengli Clinical Medical College of Fujian Medical University, Fujian Provincial Hospital, Fuzhou, Fujian People’s Republic of China; 3 State Key Laboratory of Ultrasound in Medicine and Engineering, College of Biomedical Engineering, Chongqing Medical University, Chongqing, People’s Republic of China

**Keywords:** Endometriosis, Adolescence, Dysmenorrhea pattern, Predictor

## Abstract

**Background:**

Endometriosis (EMs) is a chronic and progressive disease that, if diagnosed late, can lead to infertility and deep infiltrating endometriosis (DIE). Dysmenorrhea is the most prominent symptom of EMs. However, limited research exists on the specific correlation between dysmenorrhea patterns and EMs. Early prevention of EMs is essential to effectively manage the progression of the disease, and is best detected during adolescence. Our objective was to associate the development of EMs with dysmenorrhea patterns during adolescence and quantify the risk of adult EMs for adolescent girls, with the aim of supporting primary intervention strategy planning.

**Methods:**

This case–control study examined predictors for adult EMs based on dysmenorrhea patterns in adolescents. We collected 1,287 cases of 641 EMs and 646 healthy females regarding their basic demographic information, adolescent menstrual characteristics, adolescent dysmenorrheal patterns, and adolescent lifestyles. Age-matching (1-to-1) was employed to control for the confounding effect of age between the groups. Least Absolute Shrinkage and Selection Operator (LASSO) and logistic regression models were utilized to identify predictors for adult EMs. The predictive value of the model was evaluated using the area under the receiver operating characteristic curve (AUC) and the C-index, while Hosmer–Lemeshow Test assessed the goodness of fit of the model. Data from one additional cohort in Shenzhen hospitalized with EMs were used to external validation were analyzed.

**Results:**

Individuals who always experienced dysmenorrhea had a risk of adult endometriosis 18.874 (OR = 18.874; 95%CI = 10.309–34.555) times higher than those occasional dysmenorrhea, The risk of developing EMs was 5.257 times higher in those who experienced dysmenorrhea more than 12 months after menarche than in those who experienced dysmenorrhea less than 6 months after menarche (OR = 5.257, 95% CI = 3.343–8.266), AUC in the external validation cohort was 0.794(95%CI: 0.741–0.847). We further found that high-intensity physical activity and sun-sensitive skin of burning were influential factors in high-frequency dysmenorrhea. The AUC value for the internal evaluation of the model was 0.812 and the AUC value for the external validation was 0.794.

**Conclusion:**

Our findings revealed that the frequency of dysmenorrhea during adolescence contributed to the development of adult endometriosis. The frequency and onset of dysmenorrhea in adolescence were promising predictors for adult EMs. Both internal and external validation proved the model's good predictive ability.

**Trial registration:**

http://www.chictr.org.cn/, TRN: ChicTR2200060429, date of registration: 2022/06/01, retrospectively registered.

**Supplementary Information:**

The online version contains supplementary material available at 10.1186/s12889-024-17825-2.

## Introduction

Endometriosis (EMs) is a chronic inflammatory disease defined by the presence of endometrial tissue outside the uterine cavity [1]. Approximately 10% of reproductive women are diagnosed with EMs worldwide, that is about 190 million women worldwide who are affected with EMs, according to the 2017 population estimation generated by the World Bank [2, 3].The main clinical manifestations of EMs are menstruation-related pain, and dysmenorrhea and infertility may also occur in patients [4].

Previous studies have investigated risk factor profiles for EMs, but there are very few modifiable risk factors. In addition, despite the robust evidence of symptom onset during adolescence and young adulthood for most adult women with EMs, few studies exist focusing on adolescents and young adults.

Although the pathogenies of EMs are unclear, more studies suggest EMs begin early in the lifecycle, pain symptoms associated with EMs may first appear in adolescence [5]. Dysmenorrhea is the most common gynecological symptom in adolescence, with a prevalence of 50% to 90% [6]. It seriously affects adolescents' physical and mental health, such as school absenteeism, sleep disturbance, anxiety, and depression, making it an important social public health problem [7–9].

The relationship between EMs and dysmenorrhea at the adolescent age remains unclear. Given the long-term cumulative negative effects of EMs on women's health, it is crucial to shift attention to EMs at an earlier stage in the life cycle. Dysmenorrhea, the most prevalent and significantly impairing menstrual symptom during adolescence, could potentially serve as one of the predictive indicators or even the sole observable symptom in the early stages of EMs.

To effectively control the disease, patients with symptomatic EMs should be detected at an early stage, preferably in adolescence. However, the association between adolescent dysmenorrhea and EMs has only been reported qualitatively, which are not adequate for the establishment of management strategies. The onset, frequency, and impact factors of adolescent dysmenorrhea pattern related to EMs should be quantified, as well as the expectation from adolescent themselves, guardians, and teachers.

We aim to identify potential risk factors during adolescence that are able to inform adult EMs and then to quantify the association between adolescent dysmenorrhea patterns and adult EMs. It is conducive to the early prevention and intervention of diseases, stratification of patients, and individualized management to reduce disease symptoms at the critical windows of exposure, change disease development, and lower fertility disruption rate.

## Materials and methods

This is a case–control study, which evaluates risk factors for adult EMs based on the adolescent dysmenorrhea pattern and lifestyle during adolescents. This study was approved by the Medical Ethics Committee of Shenzhen Maternity and Child Healthcare Hospital (LLYJ2022-112–050). The study conformed to the tenets of the Declaration of Helsinki. The need for written informed consent to participate was waived by the Medical Ethics Committee of Shenzhen Maternity and Child Healthcare Hospital due to the retrospective nature of the study.

### Study population

The required information was collected for females at Shenzhen Maternity & Child Healthcare Hospital, from July 2019 to February 2022.

Inclusion criteria for the case group consisted of patients diagnosed with EMs through pathological examination after undergoing surgical treatment on our hospital. The control group was composed of women who had regular physical examinations in our hospital examination center. The definition of "healthy" was that the women who participated in the physical examination did not have any abnormalities in all gynecological examination items (including B-ultrasound, gynecological examination, gynecological-related blood biochemical examination, etc.), and were clearly diagnosed with no uterine fibroids, adenomyosis, endometriosis, infertility, or other diseases that may be related to the study. The two groups were aged 18 to 55.

**Fig. 1:**
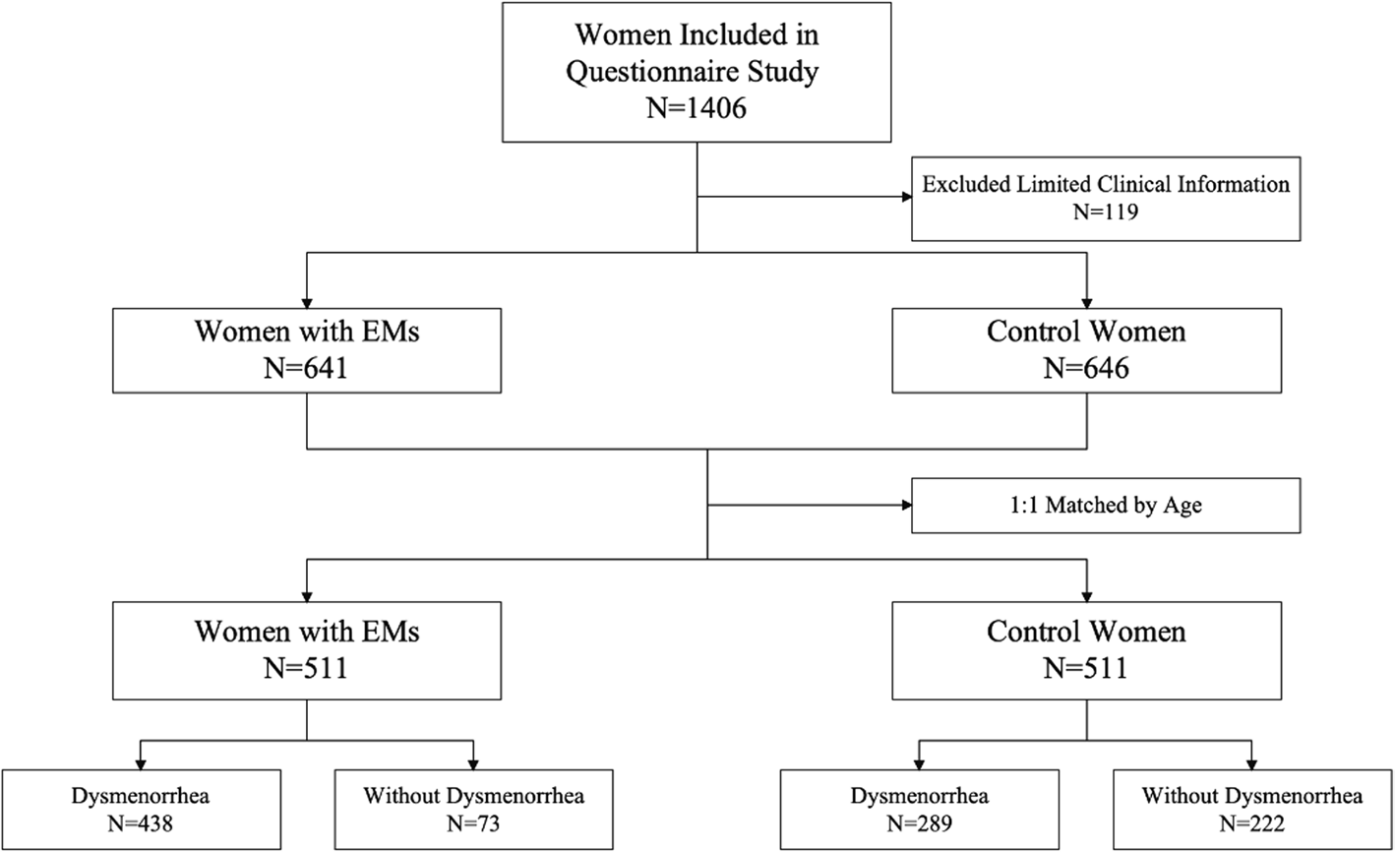
The procedure in the study. We enrolled 1406 patients. 119 cases were excluded and 1287 cases were finally included in the study. 641 EMs cases and 646 controls were analyzed. The age-matching generated 1022 data

A total of 1406 females were recruited. Of these, 32 were excluded due to incorrectly completed questionnaires, 87 were excluded without satisfying the inclusion criteria, 73 were removed from the case group, and 46 from the control group. 1287 females were finally included, with 91.5% of valid questionnaires, of which 641 were from the case group and 646 were from the control group (Fig. 1).

The data collection methods of the two groups are different: The telephone questionnaire survey was targeted at EMs patients, who received questionnaires over the phone; the WeChat survey was targeted at women who have undergone physical examination in our physical examination department, and they fill out the questionnaire on WeChat.

### Questionnaire

We designed a questionnaire(eAppendix 1 in Supplement) via literature search and qualitative research, which consists of basic demographic information, physiological characteristics during adolescence, lifestyle, dietary habits, and family members, with a total of 22 questions, each set with clear options. The questionnaire has completed a pre-survey of 53 people, and the expression of individual items has been optimized through the pre-survey(eAppendix 2 in Supplement).

Dysmenorrhea pattern -related questions were:

1. Frequency of dysmenorrhea: Occasional dysmenorrhea is defined as dysmenorrhea less than 6 months in a year; frequent dysmenorrhea is defined as menstrual pain greater than 6 months less than 12 months in a year. Always dysmenorrhea is dysmenorrhea with every menstrual period.
2. Degree of dysmenorrhea: mild pain was defined as not interfering with sleep; moderate pain was defined as slightly interfering with sleep; and severe pain was defined as awakening during sleep.
3. Degree of distress for dysmenorrhea: the scale is 0–10, corresponding to the degree of no to very distressed/depressed: A score of 0 means no worries, and distress is defined as the score.
4. First time of dysmenorrhea: We classify it as within 6 months of menarche, between 6 and 12 moths, and over 12 months.
5. Unexplained abdominal pain during non-menstrual period.
6. Family history of dysmenorrhea: It is divided into same generation (close sisters/cousins), previous generation (mother/mother's close sisters), and intergenerational (grandmother).
7. Age of Menarche: ≤ 11 years old, between 12 to 16 years old, > 16 years old.
8. Menstrual Cycle: < 21 days, between 21 to 26 days, between 27 to 35 days, > 35 days.
9. Duration of Menstruation: ≤ 4 days, between 5 to 8 days, > 8 days.

Lifestyle is evaluated via items as follows:

1. The intensity of physical activities: high-intensity (aerobics, swimming, basketball, etc.), medium-intensity (running, cycling, etc.), low-intensity (jogging, Tai Chi, etc.), and light intensity (walking, radio gymnastics, etc.).
2. Sun-exposed skin sensitivity condition refers to exposure to the sun for more than 2 h without sun protection.
3. Amount of dairy consumed per day: ≤ 1 portion, > 1 portion.
4. Hours of sleep per day: ≥ 8 h, < 8 h; 5. Dietary structure: Predominantly red meat (pork, beef and lamb), Predominantly white meat (chicken, duck and fish), vegetable-based.

### Statistical analysis

All statistical analysis were performed using *SPSS 25.0* (*SPSS*, Chicago, IL, USA) and* R* (version 4.2.2) software with packages "readxl", “dplyr”, “plyr”, “rms”, “pROC”,“nomogramEx” “glmnet”and “ggplot2”, using functions predict (model, dataset,type = c ("response")). We presented the demographic, social-economical and characteristics of the case and control groups. Continuous variables with normal distribution were described by means and standard deviations, medians and quartiles described variables without normal distribution. Categorical variables were described by frequency and rate (%). T-test was performed testing differences between groups for continuous variables with normal distribution and Wilcoxon rank sum test for continuous variables without normal distribution. Categorical variables were tested by chi-square test. The main advantage of LASSO regression is that it can select the most relevant variables by processing multiple dependent variables, resulting in a simpler model, Logistic regression and LASSO (Least Absolute Shrinkage and Selection Operator) regression are commonly used variable screening methods, before we chose LASSO, we compared the performance of them. The results showed that the overall performance of the "LASSO regression" was better (eAppendix 3 in Supplement). The potential risk factors for EMs were identified by LASSO-logistic regression. None-zero variables in lasso regression were included in the univariate logistic analysis. Variables with *p* < 0.05 in the univariate logistic analysis were subsequently included in the multivariate logistic analysis. A multivariate logistic regression was used to construct a nomogram model to predict the occurrence of EMs. Independent predictors (*p* < 0.05) were assessed by a multivariate logistic regression and then recruited to develop the nomogram using the data for predicting the occurrence EMs. Predictor lines were drawn upward to confirm the points received from the nomogram. The sum of these points was located on the “Total Points” axis; subsequently, a line was drawn downward to project on the bottom scales, which determined the possibility of EMs. Thereafter, the visual prediction model was externally validated. The Hosmer–Lemeshow test was used to assess the goodness of fit of the model. The AUC, C-index were used to evaluate the predictive accuracy and conformity. All statistical tests were considered significant when two-tailed *p* < 0.05.

## Results

### Characteristics related to dysmenorrhea in selected patients

Total 1287 participants were analyzed, including 641 women with EMs and 646 health controls. To avoid recall bias caused by the age difference, matching was performed by age ± one year, and a total of 511 pairs were obtained (Table 1). Age of menarche, frequency of dysmenorrhea, onset of dysmenorrhea, degree of dysmenorrhea, degree of dysmenorrhea distress, family history of dysmenorrhea, intensity of physical activity, daily intake, sun-sensitivity skin, sleep duration, dietary structure, all of the above variables were statistically different between the two groups(*p* < 0.05). No statistical differences are in the two groups with pelvic pain of non-menstrual period, menstrual cycle and Duration of Menstruation. All collected variables were from the patients' adolescence period.

**Table 1:** Characteristics in women with or without EMs matched by age

Characteristic	Control *n* = 511	EMs *n* = 511	*p* value
Age(years)	31.85 ± 5.51	31.68 ± 5.46	0.612
Age at Diagnosis(years)	-	30.90 ± 5.31	
Age of Menarche (years)			< 0.001*
≤ 11	41(8.0%)	184(36.0%)	
12–16	424(83.0%)	309(60.5%)	
> 16	46(9.0%)	18(3.5%)	
Menstrual Cycle (days)			0.192
< 21	14(2.7%)	13(2.5%)	
21–26	125(24.5%)	156(30.5%)	
27–35	345(67.5%)	318(62.2%)	
> 35	27(5.3%)	24(4.7%)	
Duration of Menstruation (days)			0.143
≤ 4	82(16.0%)	105(20.5%)	
5–8	414(81.0%)	395(77.3%)	
> 8	15(2.9%)	11(2.2%)	
Dysmenorrhea Frequency			< 0.001*
Never	222(43.4%)	73(14.3%)	
Occasionally	214(41.9%)	122(23.9%)	
Often	60(11.7%)	166(32.5%)	
Always	15(2.9%)	150(29.4%)	
Onset of Dysmenorrhea			< 0.001*
Within 6 months of menarche	317(62.0%)	132(25.8%)	
6—12 months after menarche	56(11.0%)	35(6.8%)	
12 months after menarche	138(27.0%)	344(67.3%)	
Degree of Dysmenorrhea			< 0.001*
Mild	346(67.7%)	148(29.0%)	
Moderate	104(20.4%)	166(32.5%)	
Severe	61(11.9%)	197(38.6%)	
Degree of dysmenorrhea distress	4.20 ± 2.78	6.42 ± 2.15	< 0.001*
Pelvic Pain of Non-menstrual Period			0.811
No	415(81.2%)	412(80.6%)	
Yes	96(18.8%)	99(19.4%)	
Family History of Dysmenorrhea			< 0.001*
No	327(64.0%)	215(42.1%)	
The Same Generation	93(18.2%)	70(13.7%)	
The Previous Generation	69(13.5%)	188(36.8%)	
Parents Grandparents/ Atavism	22(4.3%)	38(7.4%)	
Intensity of Physical Activity			< 0.001*
Slight	209(40.90)	111(21.72)	
Low	106(20.74)	75(14.68)	
Moderate	153(29.94)	166(32.49)	
High	43(8.41)	159(31.12)	
Daily Intake (Per Day)			< 0.001*
≤ 1	416(81.41)	475(92.95)	
> 1	95(18.59)	36(7.05)	
Sun-sensitivity Skin			< 0.001*
No	230(45.01)	116(22.70)	
Redness	209(40.90)	205(40.12)	
Burning	56(10.96)	148(28.96)	
Blisters	16(3.13)	42(8.22)	
Sleep duration			< 0.001*
≥ 8	416(81.41)	475(92.95)	
< 8	95(18.59)	36(7.05)	
Dietary structure			< 0.001*
Vegetarian diet	100(19.57)	67(13.11)	
Red diet	224(43.84)	319(62.43)	
White diet	187(36.59)	125(24.46)	
Sites of EMs			
Ovarian	0(0.0%)	315(61.6%)	
Pelvic	0(0.0%)	118(23.1%)	
Ovarian and Pelvic	0(0.0%)	78(15.3%)	
None	511(100.0%)	0(0.0%)	

^*^Significant at *p* < 0.05

### Screening for predictive factors in dysmenorrhea characteristics

**Fig. 2:**
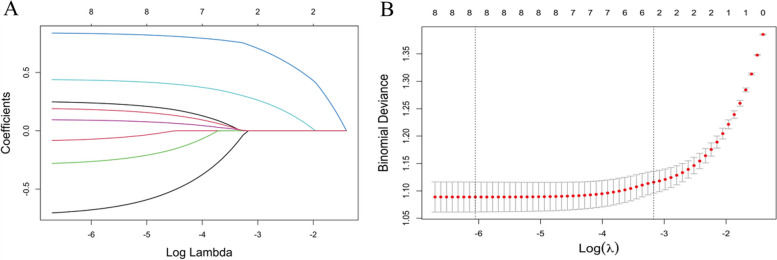
Dysmenorrhea feature selection by LASSO. **A** To differentiate EMs, LASSO regression was used for variable screening. The results showed that 2 variables were retained when the error was the smallest; that is, the place corresponding to the dotted line on the left. **B** LASSO coefficient profiles of the 8 dysmenorrhea features. A coefficient profile plot was produced against the log(λ) sequence. A vertical line was drawn at the selected optimizing value (λ), which resulted in 2 nonzero coefficients

**Fig. 3:**
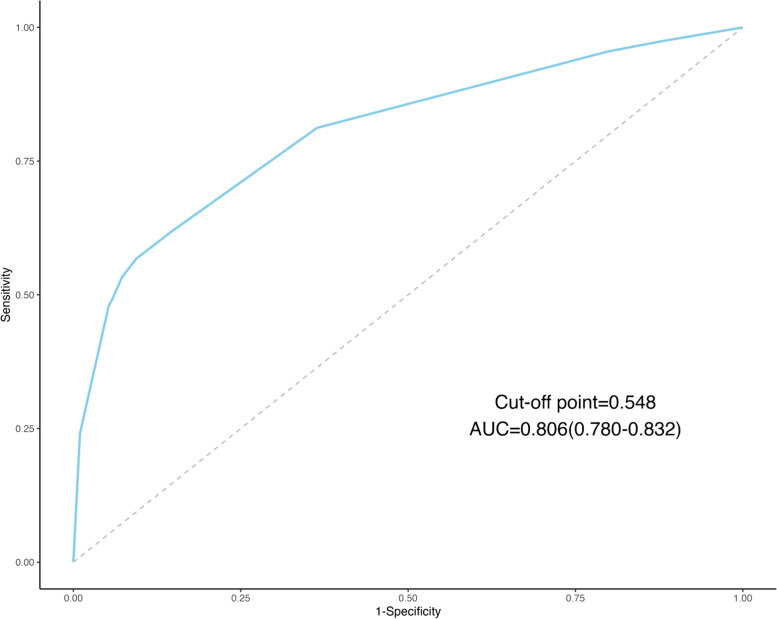
Receiver-operating Characteristic (ROC) Curves for dysmenorrhea characteristics. This AUC plot displays the performance of a model for predicting EMs in the future based on variables related to dysmenorrhea. The area under the ROC curve was 0.806 (*p* < 0.001)

We included all variables in Table 1 (excluding sites of EMs) into Lasso Regression. Figure 2 presents the results of the LASSO regression on the 9 variables included, along with their corresponding coefficients, for different values of the penalty parameter. Specially, as Log(λ) approaches -3.17 (λ is 0.042), dysmenorrhea frequency and onset of dysmenorrhea confer the largest signal in the model (Table 2). We selected variables in Table 2 into logistic regression. Univariate analysis showed that the EMs had a trend of correlation with dysmenorrhea frequency and onset of dysmenorrhea. The variables with statistical significance in the univariate analysis were subjected to multivariable logistic regression. Those who reported high frequent dysmenorrhea (often) at adolescence were more likely to have EMs (OR = 3.194; 95%CI = 1.931–5.283) than those without dysmenorrhea, the high frequent dysmenorrhea (always) at adolescence were more likely to have EMs (OR = 10.118; 95%CI = 5.193–19.711) than those without dysmenorrhea. The risk of dysmenorrhea occurring more than 12 months after menarche was 5.257 (95%CI = 3.343–8.266) times higher than the risk of developing EMs within 12 months after menarche (Table 3). The AUC was 0.806(95%CI 0.780–0.832 (*p* < 0.001)), indicating a significant performance of the risk of Ems (Fig. 2). When the cut-off point was set as 0.548, the sensitivity (0.568) and specificity (0.902) can be optimized, the positive predictive value (PPV) is 0.858, and the negative predict value (NPV) is 0.677 (Fig. 3). In addition, The Hosmer − Lemeshow test demonstrated that the model was a good fit (*p* = 0.900 > 0.05).

**Table 2:** Dysmenorrhea variables screened by LASSO regression

Characteristic	Coefficient
Age of Menarche (years)	0
Menstrual Cycle (days)	0
Duration of Menstruation (days)	0
Dysmenorrhea Frequency	0.737
Onset of Dysmenorrhea (months)	0.288
Degree of Dysmenorrhea	0
Pelvic Pain of Non-menstrual Period	0
Family History of Dysmenorrhea	0

**Table 3:** Factors Related to EMs by Logistic Regression

Variables	Univariate		Multivariate	
	OR (95%CI)	*p* value	OR (95%CI)	*p* value
Dysmenorrhea Frequency				
Never	Ref		Ref	
Occasionally	1.734(1.227–2.449)	0.002*	0.536(0.319–0.902)	0.069
Often	8.414(5.661–12.506)	< 0.001*	3.194(1.931–5.283)	< 0.001*
Always	30.411(16.807–55.025)	< 0.001*	10.118(5.193–19.711)	< 0.001*
Onset of Dysmenorrhea				
Within 6 months of menarche	Ref		Ref	
6-12 months after menarche	1.501(0.939–2.398)	0.089	1.335(0.724–2.464)	0.355
12 months after menarche	5.986(4.510–7.946)	< 0.001*	5.257(3.343–8.266)	< 0.001*

^*^Significant at *p* < 0.05

### Dysmenorrhea pattern of patients with dysmenorrhea

To explore the lifestyle habits and dysmenorrhea characteristics during adolescence of patients with EMs in the population with dysmenorrhea, we only analyzed the dysmenorrhea group (Table 4). BMI, age of menarche, menstrual cycle, frequency of dysmenorrhea, onset of dysmenorrhea, degree of dysmenorrhea, pelvic pain of non-menstrual period, family history of dysmenorrhea, whether family members smoking, the intensity of physical activity, number of servings of dairy products, skin sensitivity after sun exposure, sleep duration, dietary structure, all of these variables were statistically different between the two groups(*p* < 0.05). No statistical differences are in the two groups with menstrual cycle and duration of menstruation.

**Table 4:** Characteristics of dysmenorrhea women

Characteristic	Control *n* = 289	EMs *n* = 438	*p* value
Age(years)	30.80 ± 95	31.40 ± 5.55	0.159
Age of Menarche (years)			< 0.001*
≤ 11	30(10.4%)	167(38.1%)	
12–16	237(82.0%)	258(58.9%)	
> 16	22(7.61%)	13(3.0%)	
Menstrual Cycle (days)			0.468
< 21	8(2.8%)	12(2.7%)	
21–26	72(24.9%)	131(29.9%)	
27–35	195(67.5%)	271(61.9%)	
> 35	14(4.8%)	24(5.5%)	
Duration of Menstruation (days)			0.145
≤ 4	44(15.2%)	90(20.5%)	
5–8	236(81.7%)	339(77.4%)	
> 8	9(3.1%)	9(2.1%)	
Dysmenorrhea Frequency			< 0.001*
Occasionally	214(74.0%)	122(27.9%)	
Often	60(20.8%)	166(37.9%)	
Always	15(5.2%)	150(34.2%)	
Onset of Dysmenorrhea (months)			< 0.001*
< 6	95(32.9%)	59(13.5%)	
6–12	56(19.4%)	35(8.0%)	
> 12	138(47.8%)	344(78.5%)	
Degree of Dysmenorrhea			< 0.001*
Mild	124(42.9%)	75(17.1%)	
Moderate	104(36.0%)	166(37.9%)	
Severe	61(21.1%)	197(45.0%)	
Pelvic Pain of Non-menstrual Period			0.012*
No	201(69.6%)	342(78.1%)	
Yes	88(30.4%)	96(21.9%)	
Family History of Dysmenorrhea			< 0.001*
No	145(50.2%)	163(37.2%)	
The Same Generation	66(22.8%)	61(13.9%)	
The Previous Generation	58(20.1%)	178(40.6%)	
Parents Grandparents/ Atavism	20(6.9%)	36(8.3%)	
Sites of EMs			
Ovarian	0(0.0%)	264(60.3%)	
Pelvic	0(0.0%)	103(23.5%)	
Ovarian and Pelvic	0(0.0%)	71(16.2%)	
None	289(100.0%)	0(0.0%)	

^*^Significant at *p* < 0.05

### Screening for predictive factors in dysmenorrhea patients

**Fig.4:**
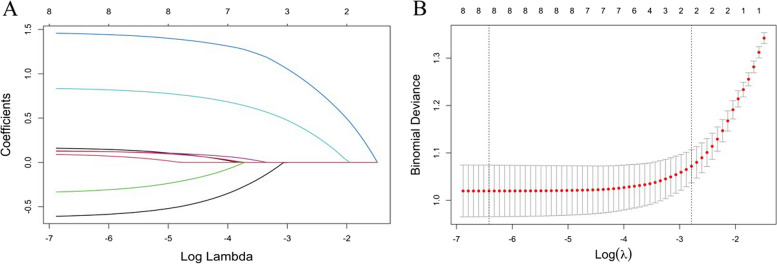
Dysmenorrhea feature in patients with dysmenorrhea selection by LASSO. **A** To differentiate EMs in patients with dysmenorrhea, LASSO regression was used for variable screening. The results showed that 2 variables were retained when the error was the smallest; that is, the place corresponding to the dotted line on the left. **B** LASSO coefficient profiles of the 8 dysmenorrhea features. A coefficient profile plot was produced against the log(λ) sequence. A vertical line was drawn at the selected optimizing value (λ), which resulted in 2 nonzero coefficients

**Fig. 5:**
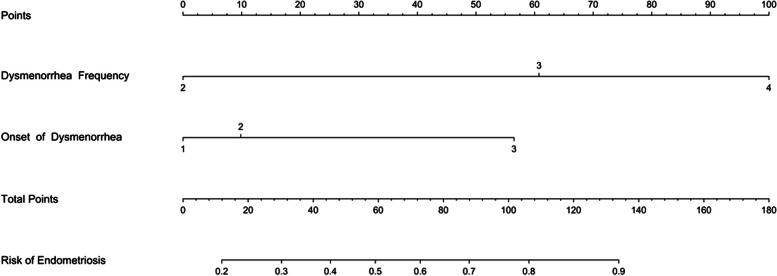
Nomogram for predicting the risks of EMs in person with dysmenorrhea. This nomogram was developed to predict the probability of developing EMs in person with dysmenorrhea. The nomogram includes two predictors of EMs risk: dysmenorrhea frequency and onset of dysmenorrhea. Notes: In the frequency of dysmenorrhea, 1 refers to never experiencing dysmenorrhea; 2 refers to occasionally; 3 refers to often; 4 refers to always. In the onset of dysmenorrhea, 1 refers to within 6 months after menarche; 3 refers to more than 12 months; 2 refers to between them

**Fig. 6:**
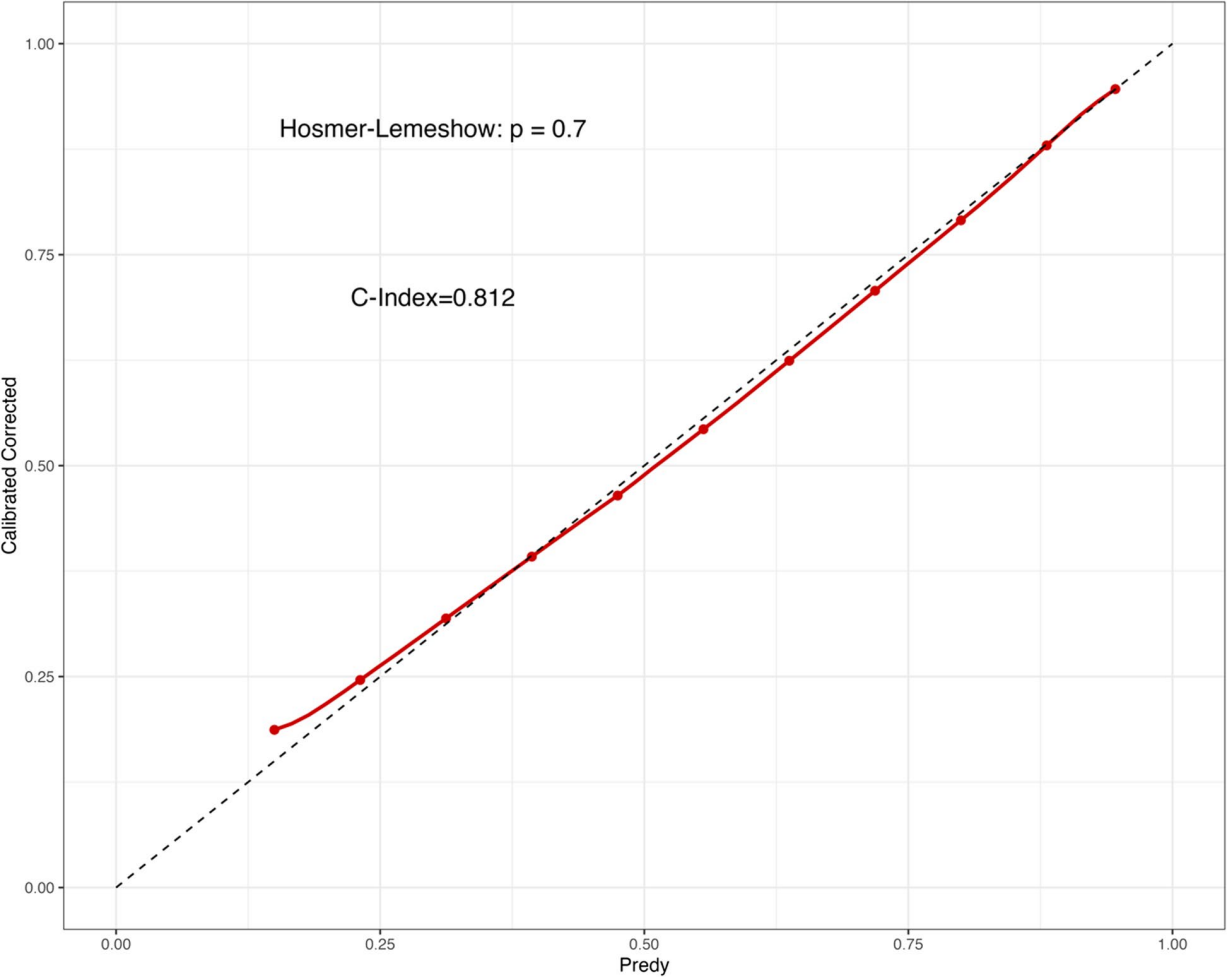
Calibration curve

**Fig. 7:**
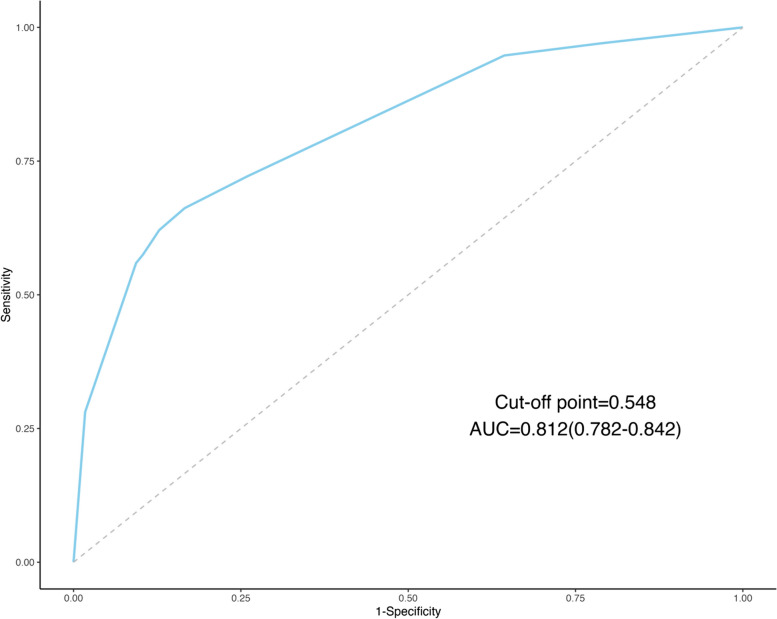
Receiver-operating Characteristic (ROC) Curves for dysmenorrhea characteristics in person with dysmenorrhea. This AUC plot displays the performance of a model for predicting EMs in person with dysmenorrhea in the future based on variables related to dysmenorrhea. The area under the ROC curve was 0.812 (*p* < 0.001)

We included all patients with dysmenorrhea (with or without EMs) in the analysis. Firstly, we included variables related to dysmenorrhea into LASSO regression. As Log(λ) approaches -2.79 (λ is 0.0615), dysmenorrhea frequency and onset of dysmenorrhea confer the largest signal in the model (Table 5, Fig. 4). The two nonzero variables were enrolled into logistic regression. Univariate analysis showed that the EMs in dysmenorrhea population had a trend of correlation with dysmenorrhea frequency and onset of dysmenorrhea. The variables with statistical significance in the univariate analysis were subjected to multivariable logistic regression. Individuals who reported high frequent dysmenorrhea (often) at adolescence were more likely to have EMs (OR = 5.959; 95%CI = 3.970–8.944) than those dysmenorrhea(Occasionally), the high frequent dysmenorrhea (always) at adolescence were more likely to have EMs (OR = 18.874; 95%CI = 10.309–34.555) than those dysmenorrhea(Occasionally), the risk of developing EMs was 5.257 times higher (95%CI = 3.343–8.266) for those who experienced dysmenorrhea more than 12 months after menarche (Table 6). The logistic regression model was constructed based on four factors, after which these two factors from the logistic regression model were integrated to the nomogram(C-Index = 0.812) ( Figs. 5 and 6). The AUC was 0.812(CI 0.782–0.842 (*p* < 0.001)), indicating a significant performance of the risk of Ems (Fig. 7). When the cut-off point was set as 0.548, the sensitivity (0.662) and specificity (0.834) can be optimized, the positive predictive value (PPV) is 0.858, and the negative predict value (NPV) is 0.620. In addition, The Hosmer − Lemeshow test demonstrated that the model was a good fit (*P* = 0.7 > 0.05).

**Table 5:** Dysmenorrhea characteristics screened by LASSO regression in dysmenorrhea subjects

Characteristic	Coefficient
Age of Menarche (years)	0
Menstrual Cycle (days)	0
Duration of Menstruation (days)	0
Dysmenorrhea Frequency	0.962
Onset of Dysmenorrhea (months)	0.404
Degree of Dysmenorrhea	0
Pelvic Pain of Non-menstrual Period	0
Family History of Dysmenorrhea	0

**Table 6:** Significant related to dysmenorrhea characteristics by logistic regression in dysmenorrhea subjects

Variables	Univariate		Multivariate	
	OR (95%CI)	*p* value	OR (95%CI)	*p* value
Dysmenorrhea Frequency				
Occasionally	Ref		Ref	
Often	4.853 (3.353–7.023)	< 0.001*	5.959(3.97–8.944)	< 0.001*
Always	17.541(9.867–31.184)	< 0.001*	18.874(10.309–34.555)	< 0.001*
Onset of Dysmenorrhea (months)				
< 6	Ref		Ref	
6–12	1.006(0.591–1.715)	0.981	1.335(0.724–2.464)	0.355
> 12	4.014(2.744–5.870)	< 0.001*	5.257(3.343–8.266)	< 0.001*

^*^Significant at *p* < 0.05

### External validation

To validate the generalizability of EMs risk score, we used data from another hospital(Luohu District Maternity and Child Healthcare Hospital) in Shenzhen, A total of 400 cases between January 2022 to June 2022 were included in the data, 200 in each of the EMs and control groups. The variables required for calculating the EMs risk score from the validation cohort were collected and the risk scores were calculated in the same way as the study cohort described.

**Fig. 8:**
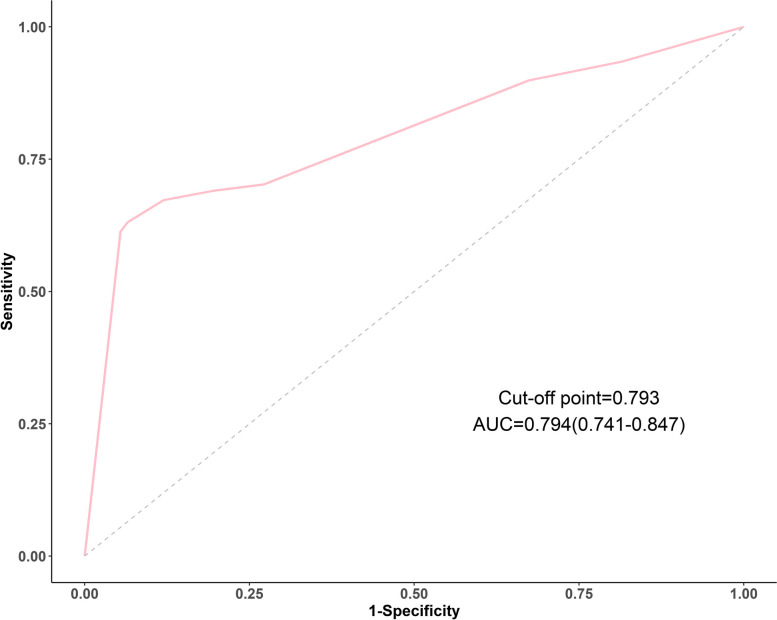
Receiver-operating Characteristic (ROC) Curves for validation cohort with dysmenorrhea. This AUC plot displays t external validation of model performance. The area under the ROC curve was 0.794 (*p* < 0.001)

For the validation cohort of EMs, 168 (84%) had dysmenorrhea in the EMs group and 92 (46%) had dysmenorrhea in the control group. The variables used in the risk score of EMs in the validation cohort are shown in Table 7. The accuracy of the risk score of EMs in the validation cohort was similar to that of the study cohort. The AUC in the validation cohort of 0.794 (95%CI: 0.741–0.847) (Fig. 8),Hosmer and Lemeshow Test X2 = 10.852, df = 8, *p*-value = 0.210 (*p* > 0.05), both of which show good model calibration, C-index = 0.794, which suggests good model discrimination.

**Table 7:** Demographics and clinical characteristics of persons in validation cohorts

Characteristic	Control *n* = 92	EMs *n* = 168	*p* value
Age(years)	33.1 ± 6.25	35.1 ± 6.69	0.013
Dysmenorrhea Frequency			< 0.001*
Occasionally	67(72.83%)	50(29.76%)	
Often	17(18.48%)	67(39.88%)	
Always	8(8.70%)	51(30.36%)	
Onset of Dysmenorrhea			< 0.001*
Within 6 months of menarche	29(31.52%)	20(11.90%)	
6—12 months after menarche	21(22.83%)	12(7.14%)	
12 months after menarche	42(45.65%)	136(80.95%)	

^*^Significant at *p* < 0.05

### Screening for Lifestyle factors in EMs patients for dysmenorrhea frequency

**Fig. 9:**
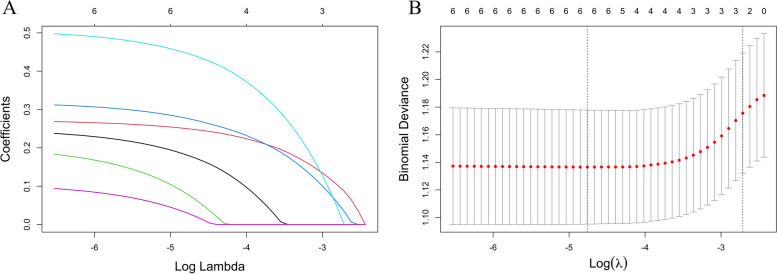
Dysmenorrhea and Lifestyle Features in Patients with Dysmenorrhea for Dysmenorrhea Frequency Selection by LASSO. **A** To differentiate frequency of dysmenorrhea in patients with dysmenorrhea, LASSO regression was used for variable screening. The results showed that 2 variables were retained when the error was the smallest; that is, the place corresponding to the dotted line on the left. **B** LASSO coefficient profiles of the 6 lifestyle features. A coefficient profile plot was produced against the log(λ) sequence. A vertical line was drawn at the selected optimizing value (λ), which resulted in 2 nonzero coefficients

We included patients with EMs in the analysis. And, according to the frequency of dysmenorrhea (High frequency dysmenorrhea including often and always dysmenorrhea; Low frequency dysmenorrhea including occasionally), we categorized patients with EMs into low-frequency and high-frequency pain groups, aiming to investigate factors influencing the frequency of dysmenorrhea in the population with EMs. Firstly, we included lifestyle variables related to dysmenorrhea into LASSO regression. As Log(λ) approaches -2.71 (λ is 0.066), intensity of physical activity and sun-sensitivity-skin confer the largest signal in the model (Table 8, Fig. 9). The two nonzero variables were enrolled into logistic regression. The variables with statistical significance in the univariate analysis were subjected to multivariable logistic regression. Individuals with high-intensity physical activity had 2.886 (95% CI = 1.525–5.464) times higher risk of developing EMs compared to those with slight activity. Those with sun-sensitive skin of burning had a 2.010 (95% CI = 1.076–3.752) times higher risk of developing EMs compared to those without  (Table 9).

**Table 8:** Characteristics screened by LASSO regression in dysmenorrhea subjects for dysmenorrhea frequency

Characteristic	Coefficient
Exposure to smoking	0
Intensity of Physical Activity	0.087
Dairy Intake	0
Sun-Sensitivity Skin	0.034
Sleep Duration	0
Dietary preference	0

**Table 9:** Factors related to dysmenorrhea frequency in dysmenorrhea subjects

Variables	Univariate		Multivariate	
	OR (95%CI)	*p* value	OR (95%CI)	*p* value
Intensity of Physical Activity				
Light intensity	Ref		Ref	
Low-intensity	1.559(0.808–3.007)	0.186	1.340(0.683–2.629)	0.394
Medium-intensity	1.724(0.995–2.986)	0.052	1.570(0.897–2.748)	0.114
High	3.609(1.956–6.660)	< 0.001*	2.886(1.525–5.464)	0.001*
Sun-sensitivity Skin				
No	Ref		Ref	
Redness	1.105(0.657–1.858)	0.707	0.970(0.569–1.653)	0.911
Burning	2.555(1.400–4.663)	0.002*	2.010(1.076–3.752)	0.028*
Blisters	3.094(1.183–8.088)	0.021*	2.258(0.840–6.072)	0.106

^*^Significant at *p* < 0.05

## Discussion

This study characterized dysmenorrhea pattern during adolescent at patients with EMs. The significant association between dysmenorrhea frequency during adolescent and adult EMs provided a feasible approach to identify population developing EMs in the future. Onset time of dysmenorrhea was also a predictor for EMs. Additionally, we developed a model based on dysmenorrhea pattern during adolescent for distinguishing individuals at high risk for EMs among cohorts with dysmenorrhea. Our study is distinctive in that we started earlier in the life cycle and explored risk factors based on dysmenorrhea patterns during adolescent for the future development of EMs.

The precise role of adolescent dysmenorrhea characteristics in the pathogenesis of EMs remains uncertain. We found an increased risk of developing EMs among individuals who experience dysmenorrhea during adolescence. These findings are consistent with the findings of Clemenza et al. [10]. The increased uterine contractions leading to dysmenorrhea, coupled with the increased chances of endometrial tissue entering the pelvic cavity, suggest that dysmenorrhea may be a precursor to EMs [11]. Currently, research on dysmenorrhea associated with EMs primarily focuses on understanding its mechanisms and severity, while studies examining the patterns of dysmenorrhea are scarce. Only one study mentioned the frequency of dysmenorrhea related to EMs, indicating that pain is commonly experienced by EMs patients (83.3%, *n* = 273) [12]. Our study revealed that the frequency of dysmenorrhea is an independent risk factor for predicting EMs with high predictive efficacy. The frequency of dysmenorrhea (always) was found to be nearly 19 times higher compared to individuals without EMs.

The pain associated with EMs is caused by the interplay of multiple factors [13, 14]. Many pain mediators have been found to be associated with the occurrence of dysmenorrhea in EMs, and they may directly induce excitatory inward currents or alter the function of ion channels in the uterine smooth muscle and vasculature, leading to uterine ischemia, hypoxia, and uterine spasm-pain [15–18]. In patients with EMs, cytokines(IL-1β, IL-6, and TNF-α), growth factors(NGF and VEGF) and chemokines are upregulated to participate in peripheral neural sensitization or amplify inflammatory responses in the microenvironment, resulting in dysmenorrhea [19, 20]. And, the emergence of new nerves and blood vessels in the lesion sites is also one of the contributing factors to the occurrence of dysmenorrhea [21], Additionally, EMs tissue can promote the expression of neurotrophic factors (NGF, BDNF, NT4, and NT5) to regulate pain associated with EMs [22, 23].The causal relationship between the frequency of dysmenorrhea and EMs remains unclear. The presence of active ectopic endometrial almost always leads to dysmenorrhea, whereas the frequency of dysmenorrhea unrelated to EMs is sporadic. However, it is also possible that the higher the frequency of dysmenorrhea potentially increase the risk of active endometrial implants. The two may be cause and effect of each other. If dysmenorrhea frequency is used as the primary screening indicator for identifying adolescents at risk of developing EMs, it would significantly reduce the number of individuals requiring focused management.

Furthermore, our research has found that the onset of dysmenorrhea occurring for the first time after 12 months of menarche is another high-risk factor for EMs. It may be associated with the repeated repair of tissue damage in EMs [24]. There is a latency period between the onset of the disease and the appearance of symptoms, which is why dysmenorrhea caused by EMs rarely occurs within the first 12 months after menarche [25]. ACOG reported that endometriosis is one of the main causes of secondary dysmenorrhea [26]. Secondary dysmenorrhea is generally defined as dysmenorrhea that occurs more than 12 months after menarche [25], our conclusions are consistent with previous studies.

In addition, we conducted analysis on high-intensity physical activity(PA) and as potential risk factors for high-frequency dysmenorrhea. By intervening these factors, we can potentially prevent the occurrence of high-frequency dysmenorrhea. Because our study was the first to find a correlation between dysmenorrhea frequency and EMs, there have been no studies correlating dysmenorrhea frequency with sun-sensitive skin of burning and high-intensity PA, but we have found a number of studies correlating these two factors with EMs. Previous studies on the relationship between EMs and PA are controversial. Ricci et al. found PA may affect the occurrence of EMs by altering estrogen levels and ovulation frequency, high-intensity exercise induces the release of inflammatory factors (ROS, TNF-α, IL-6), causing inflammatory reactions, which may promote the occurrence of EMs [27]. But, Hemmert et al. found no significant correlation between weekly PA and EMs [28]. Therefore, the relationship between PA and EMs requires further study, our study further supports that high intensity PA is a risk factor for EMs. Kvaskoff et al. found a significantly increased risk of EMS in people susceptible to sun exposure and those with moles or freckles, and a positive dose–response relationship between EMS risk and skin sensitivity to sun exposure and the number of moles and freckles [29]. Solar UVB irradiance and 25-hydroxyvitamin D (25(OH)D) levels influence EMs development, and people who have sun-sensitive skin produce 25 (OH) D more efficiently from solar UVB radiation [30]. Skin sensitivity to sunlight exposure may be a risk factor for EMs.

In summary, our study has provided a more refined pattern of dysmenorrhea compared to previous research. We found that high frequency of dysmenorrhea during adolescence is a risk factor for EMs. Based on risk factors during adolescence, we constructed a simple and accessible prediction model to assess the early risk of developing EMs, and verified the effectiveness of the model through internal evaluation and external validation. The incidence of dysmenorrhea in adolescents is high, and it is not feasible that each adolescent with dysmenorrhea is monitored and managed for a long period of time, and it may also lead to overtreatment and high disease costs. Through our prediction model, we can accurately identify high EM risk groups of adolescents with dysmenorrhea. This could be beneficial for early identification, early intervention, reducing the progress of EMs, and minimizing long-term adverse consequences, particularly in terms of preserving fertility. It has the potential to yield favorable clinical and societal benefits.

Furthermore, in order to study the disease history of EMs throughout adolescents to adulthood, and to reduce the long-term cumulative harm caused by EMs by early diagnosis and treatment, we will be conducting research that spans more than a decade. We have established a cohort of 1035 adolescent females. Future research will use our established EMs risk assessment model to screen out adolescents at high risk of EMs, and focus on monitoring and long-term management. At the same time, molecular biology and multi-omics analyses will be conducted to further verify the assessment level of the model.

### Limitation

1. It is difficult to avoid recall bias when obtaining past information in case–control studies, but recall bias regarding pain is relatively reduced compared to other indicators due to the deep memory it generates [31, 32]. During the follow-up, we found that the vast majority of respondents had a strong impression of pain and could clearly and in detail describe the basic situation of adolescent pain. In order to control recall bias, we excluded subjects who could not accurately recall the situation during adolescence, and we described the specific indicators of dysmenorrhea patterns in detail. In addition, we introduced lifestyle of dysmenorrhea to help recall from multiple perspectives and reduce bias. Age-match could also control the bias due to the recall period caused by age between two groups. Currently, we will conduct prospective cohort study to further validate and optimize our conclusions.
2. Although we used some internal and external validation methods for this study, we still need to validate this model through extensive, multicenter, prospective studies, for a more generalized interpretation.
3. We cannot make any causal inferences due to case–control studies and we require future cohort to validate the present results. However, this study has the cause and effect in the future and provides evidence of target population identification.

## Conclusion

Our study was the first to find a correlation between dysmenorrhea frequency during adolescence and EMs, which could be a new indicator for people at high risk for EMs on earlier stage of the life cycle. The results of our study provide a high level of evidence-based medical evidence for secondary prevention of EMs and the best opportunity to interrupt the trajectory of disease symptoms and progression, including fertility protection. We will conduct prospective cohort study to further validate and optimize our conclusions in the future.

### Supplementary Information

**Additional file 1: eAppendix 1.** Adolescent menstrual Symptoms and lifestyle questionnaire. **eAppendix 2.** Pre-survey. **eAppendix 3.** Comparative analysis of Logistic and LASSO regressions. **eTable1.** Univariate and multivariate logistic regression analysis for risk of Ems. **eFigure1.** Calibration Curve (Logistic). **eFigure2**. ROC of the risk prediction model for EMs (Logistic). **eTable2. **All variables screened by LASSO regression. **eFigure3.** Dysmenorrhea Feature in Patients with Dysmenorrhea Selection by LASSO. **eTable3.** Univariate and multivariate logistic regression analysis for risk of Ems. **eFigure4.** Calibration Curve (LASSO). **eFigure5. **ROC of the risk prediction model for EMs (LASSO).

## Data Availability

The questionnaire can be found in the article/ Supplementary Material.

## Declarations

All methods were carried out in accordance with relevant guidelines and regulations.
