# Supplementary material for: Dysmenorrhea pattern in adolescences informing adult endometriosis
Source: BMC Public Health. 2024 Feb 5;24:373. doi: 10.1186/s12889-024-17825-2 (PMC10840152; doi:10.1186/s12889-024-17825-2)
Supplement: Supplementary file 1 — Additional file 1: eAppendix 1. Adolescent menstrual Symptoms and lifestyle questionnaire. eAppendix 2. Pre-survey. eAppendix 3. Comparative analysis of Logistic and LASSO regressions. eTable1. Univariate and multivariate logistic regression analysis for risk of Ems. eFigure1. Calibration Curve (Logistic). eFigure2. ROC of the risk prediction model for EMs (Logistic). eTable2. All variables screened by LASSO regression. eFigure3. Dysmenorrhea Feature in Patients with Dysmenorrhea Selection by LASSO. eTable3. Univariate and multivariate logistic regression analysis for risk of Ems. eFigure4. Calibration Curve (LASSO). eFigure5. ROC of the risk prediction model for EMs (LASSO). [file 12889_2024_17825_MOESM1_ESM.docx]

**Supplementary Online Content**

**eAppendix 1. Adolescent menstrual Symptoms and lifestyle questionnaire**

**eAppendix 2. Pre-survey**

**eAppendix 3. Comparative analysis of Logistic and LASSO regressions**

**eTable1.** Univariate and multivariate logistic regression analysis for risk of EMs

**eFigure1.** Calibration Curve（Logistic）

**eFigure2**. ROC of the risk prediction model for EMs（Logistic）

**eTable2.** All variables screened by LASSO regression

**eFigure3.** Dysmenorrhea Feature in Patients with Dysmenorrhea Selection by LASSO

**eTable3.** Univariate and multivariate logistic regression analysis for risk of EMs

**eFigure4.** Calibration Curve (LASSO)

**eFigure5.** ROC of the risk prediction model for EMs（LASSO）

**eAppendix 1.** Adolescent menstrual Symptoms and lifestyle questionnaire

Hi! This questionnaire is designed to understand your health as a teenager and all data will be kept strictly confidential. Your true answer will be of great significance to our research, thank you for your contribution!

**1. What is your age? (Just fill in the number) [Fill-in-the-blank test] ***

_________________________________

**2.** **What is your highest degree? [Single-choice test] ***

| (1) Middle school or below |
| --- |
| (2) Senior school/ Middle special school |
| (3) Junior college |
| (4) Bachelor |
| (5) Master or above |

**3.** **What is your profession? [Single-choice test] ***

| (1) Specialists (Teacher/Doctor/lawyer) |
| --- |
| (2) Service workers (Caterer/Driver/Salesperson) |
| (3) Freelancer (Writer/Artist/Photographer/Tourist) |
| (4) Worker（Factory workers/Construction workers/City sanitation workers） |
| (5) Company staff |
| (6) Institution person/Civil service/Government workers |
| (7) Student |
| (8) Housewife |
| (9) Other |

**4.** **What is your height (cm)? [Fill-in-the-blank test] ***

_________________________________

**What is your weight (Kg)？ [Fill-in-the-blank test] ***

_________________________________

**5. What is your current marital status? [Single-choice test] ***

| (1) Unmarried |
| --- |
| (2) Married |
| (3) Divorce |
| (4) Widowed |

**6.** **What is your age of menarche? [Single-choice test] ***

| (1) ≤11years old |
| --- |
| (2) 12-16 years old |
| (3) ＞16 years old |

**7.** **What was your menstrual cycle during puberty (10-19 years old) (how often did you get your period)? [Single-choice test] ***

| (1) ＜21Days |
| --- |
| (2) 21-26 Days |
| (3) 27-35 Days |
| (4) ＞35 Days |

**8.** **What was the number of days of your period (duration of each period) during puberty (10-19 years old)? [Single-choice test] ***

| (1) ≤2 Days |
| --- |
| (2) 3-4 Days |
| (3) 5-8 Days |
| (4) ＞8 Days |

**9.** **How often did you have dysmenorrhea during your adolescence (10-19 years old)? [Single-choice test] ***

| (1) Never (Please skip to the 17th question) |
| --- |
| (2) Occasionally （Dysmenorrhea less than 6 months in a year） |
| (3) Often（Greater than 6 months less than 12 months in a year） |
| (4) Always（Dysmenorrhea with every menstrual period） |

**10.** **When did you first experience dysmenorrhea? [Single-choice test] ***

| (1) Within 6 months after menarche |
| --- |
| (2) Within 6-12 months after menarche |
| (3) More than 12 months after menarche |

**11.** **What is the worst pain you can experience when you experience dysmenorrhea? [Single-choice test] ***

| (1) 0：Painless |
| --- |
| (2) 1-3：Mild pain (Without interfering with sleep) |
| (3) 4-6：Moderate pain (Slightly interferes with sleep) |
| (4) 7-9：Severe pain (Can't sleep or wake up in pain during sleep, sweating profusely, unbearable) |
| (5) 10：Most painful (Worse than death) |

**12.** **What extent do you feel distressed by dysmenorrhea (0 being no distress, in ascending order)? [Single-choice test] ***

| ○None | ○1 | ○2 | ○3 | ○4 | ○5 | ○6 | ○7 | ○8 | ○9 | ○Distressed |
| --- | --- | --- | --- | --- | --- | --- | --- | --- | --- | --- |

**13.** **Did you often experience unexplained abdominal pain and other discomfort as described below during your non-menstrual period when you were an adolescent (10-19 years old)? [Multiple-choice test] ***

| (1) Abdominal Pain |
| --- |
| (2) Frequent Urination/Urgent Urination/Painful Urination/Hematuria |
| (3) Diarrhea/Nausea/Vomiting |
| (4) Headaches |
| (5) Other _________________ |
| (6) None |

**14.** **Which of your female family members have dysmenorrhea? [Multiple-choice test] ***

| (1) Same Generation (Sister/Cousin) |
| --- |
| (2) Previous Generation (Mother, Sister-in-law/Aunt) |
| (3) Intergenerational (Grandma) |
| (4) None |

**15.** **Did any of the family members you lived with smoke when you were an adolescent (10-19 years old)? [Single-choice test] ***

| (1) Yes |
| --- |
| (2) No (Please skip to the 21st question) |

**16.** **How often did you smoke indoors (not yourself) as an adolescent (10-19 years old)? [Single-choice test] ***

| (1) Almost daily, up to 3 months and more |
| --- |
| (2) Almost daily, less than 3 months |
| (3) At least once a week, but not every day |
| (4) Less than weekly, several weeks or more apart |
| (5) Never |

**17.** **What was your most frequent physical activity intensity during your adolescence (10-19 years old)? [Single-choice test] ***

| (1) Small (Walking/Doing Broadcast Exercises) |
| --- |
| (2) Mild (Recreational Volleyball/Jogging/Tai Chi) |
| (3) Moderate (Cycling/Running/Table Tennis) |
| (4) High intensity but not lasting (Playing Badminton/Basketball/Soccer) |
| (5) High intensity and long-lasting (Sets of Aerobics Exercises/Swimming) |
| (6) None |

**18.** **How many servings of dairy products (including milk, yogurt, ice cream, cheese, shakes, butter, etc.) did you use daily during your adolescence (10-19 years old)? [Single-choice test] ***

| (1) ≤1 serving |
| --- |
| (2) 2-4 servings |
| (3) ＞4 servings |

**19.** **Do you experience any of the following conditions on your skin after sun exposure (2 hours or more without any sun protection at all) when you were an adolescent (10-19 years old)? [Single-choice test] ***

| (1) Partial redness of the skin |
| --- |
| (2) Burning sensation |
| (3) Burning sensation with pain |
| (4) Blistering skin burns |
| (5) None |

**20.** **When you were an adolescent (10-19 years old), how many hours of continuous sleep (in hours) did you get per day? [Single-choice test] ***

| ○≤1 | ○2 | ○3 | ○4 | ○5 | ○6 | ○7 | ○8 | ○9 | ○≥10 |
| --- | --- | --- | --- | --- | --- | --- | --- | --- | --- |

**21.** **What was the main structure of your diet during adolescence (10-19 years old)? [Single-choice test] ***

| (1) Red meat (Pork, Beef, Sheep, etc.) mainly |
| --- |
| (2) White meat (Chicken, Duck, Fish, etc.) mainly |
| (3) Vegetarian-based |

**22.** **What is your current health status (in terms of gynecology)? [Multiple-choice test] ***

| (1) Healthy |
| --- |
| (2) Ovarian endometriosis |
| (3) Pelvic endometriosis |
| (4) Adenomyosis |
| (5) Infertility |
| (6) Not sure |

**eAppendix 2.** Pre-survey

The questionnaire we developed for "Menstrual Symptoms and Lifestyle in Adolescence" was formed through a three-ladder design. The first ladder involved extracting key items from literature, the second ladder involved extracting items from in-depth interviews by qualitative interview study, and the third ladder was for the feasibility of a large sample survey, it is usually recommended that the questionnaire items be controlled within 25.

Before the survey, we conducted a pre-survey to evaluate the feasibility and validity of the questionnaire. It is sufficient to detect the reliability and validity of the questionnaire when the sample size of the pre-survey was between 25 and 75. A convenient sampling method was used to include 53 adults, aged 25-50 years old, with dysmenorrhea symptoms. On the basis of the original questionnaire, we added five questions to investigate the reliability and validity of the questionnaire: 1.How difficult do you think it is to fill out the questionnaire? 2. Are there any questions in the questionnaire that are not understood? 3. Please select the items that you do not understand, need to modify, or need to add, and provide your suggestions for modification. 4. Can you understand the 0-10 rating scale? 5. Does the questionnaire include all questions related to your dysmenorrhea?

The conclusion of the pre-survey is as follows:

1. Results of the survey on whether the overall content of the measurement tool is easily understandable indicate that 96.23% of women responded that they completely understood and did not need assistance or explanation from others to complete it.

2. Results of the survey on whether the dysmenorrhea related questions in the measurement tool are comprehensive indicate that 88.68% of women responded that the items were comprehensive.

3. Results of the survey on whether the 0-10 rating scale questions were understood indicate that 98.11% of women responded that they could understand and make the correct choices.

4. Supplementary option: For item 22 "What is your current gynecological health status?", which is suggested to add an option "Not sure". All respondents expressed satisfaction with the remaining items.

We found the result of the pre-test to be good and no modification was necessary.

**eAppendix 3.** Comparative analysis of logistic and LASSO regressions

Logistic regression and LASSO (Least Absoulte Shrinkage and Selection Operator) regression are commonly used variable screening methods, and we compared the effects of the models constructed by them to clarify the best screening method for this study. We performed Logistics regression (**eTable 1、eFigure 1-2**) and LASSO regression (**eTable 2-3、eFigure 3-5**) on 511 pairs（Total 1022 study populations）respectively, and The AUC, Hosmer-Lemeshow test and C-index were used to assess predictive ability and conformity of the model. Selected a more suitable model by comparing the AIC values for further study.

The AIC（Akaike Information Criterion） value of the two model indexes was compared, the smaller the AIC value, the better the overall effect of the model. The AIC value of the Logistic regression model was 1008.43.The AIC value of the LASSO-Logistic regression model was 993.00.

The results showed that the overall effect of the "LASSO Regression " was better " Logistic Regression ".

**eTable 1.** Univariate and multivariate logistic regression analysis for risk of EMs

|  | Univariate | | Multivariate | |
| --- | --- | --- | --- | --- |
|  | OR（95%CI） | *P* value | OR（95%CI） | *P* value |
| Age（Years） | 0.994（0.972-1.017） | 0.611 |  |  |
| BMI |  |  |  |  |
| 18.5~24.0 | Ref. |  | Ref. |  |
| ＜18.5 | 2.480（1.819-3.383） | <0.001* | 1.726（1.155-2.579） | 0.008 |
| 24.0~28.0 | 0.803（0.510-1.265） | 0.345 | 1.11（0.641-1.923） | 0.709 |
| ≥28.0 | 0.199（0.024-1.661） | 0.136 | 0.36（0.039-3.304） | 0.366 |
| Marital Status |  |  |  |  |
| Unmarried | Ref. |  |  |  |
| Married | 1.314（0.996-1.733） | 0.053 |  |  |
| Others | 0.970（0.372-2.531） | 0.950 |  |  |
| Age of Menarche（Years） |  |  |  |  |
| 12~16 | Ref. |  | Ref. |  |
| ≤11 | 6.158（4.258-8.905） | <0.001* | 1.689（1.057-2.701） | 0.028 |
| ＞16 | 0.537（0.305-0.944） | 0.031* | 0.874（0.445-1.716） | 0.696 |
| Menstrual Cycle（days） |  |  |  |  |
| ＜21 | Ref. |  |  |  |
| 21~26 | 1.344（0.610-2.964） | 0.464 |  |  |
| 27~35 | 0.993（0.460-2.144） | 0.985 |  |  |
| ＞35 | 0.957（0.376-2.436） | 0.927 |  |  |
| Duration of Menstruation（days） |  |  |  |  |
| ≤4 | Ref. |  |  |  |
| 5~8 | 0.745（0.541-1.026） | 0.072 |  |  |
| ＞8 | 0.573（0.250-1.313） | 0.188 |  |  |
| Dysmenorrhea Frequency |  |  |  |  |
| Never | Ref. |  | Ref. |  |
| Occasionally | 1.734（1.227-2.449） | 0.002* | 0.691（0.382-1.250） | 0.222 |
| Often | 8.414（5.661-12.506） | <0.001* | 2.672（1.363-5.236） | 0.004 |
| Always | 30.411（16.807-55.025） | <0.001* | 7.420（3.13-17.592） | <0.001^a^ |
| Onset of Dysmenorrhea（Months） |  |  |  |  |
| ＜6 | Ref. |  | Ref. |  |
| 6~12 | 1.501（0.939-2.398） | 0.089 | 1.153（0.612-2.174） | 0.659 |
| ＞12 | 5.986（4.510-7.946） | <0.001* | 3.586（2.218-5.797） | <0.001^a^ |
| Degree of Dysmenorrhea |  |  |  |  |
| Mild | Ref. |  | Ref. |  |
| Moderate | 3.732（2.732-5.096） | <0.001* | 0.985（0.611-1.586） | 0.949 |
| Severe | 7.550（5.343-10.668） | <0.001* | 1.207（0.686-2.124） | 0.514 |
| Degree of Dysmenorrhea distress | 1.422（1.330-1.519） | <0.001* |  |  |
| Pelvic Pain of Non-menstrual Period |  |  |  |  |
| No | Ref. |  |  |  |
| Yes | 1.039（0.760-1.419） | 0.811 |  |  |
| Family History of Dysmenorrhea |  |  |  |  |
| No | Ref. |  | Ref. |  |
| The same Generation | 1.145（0.803-1.632） | 0.455 | 0.986（0.634-1.534） | 0.951 |
| The Previous Generation | 4.144（2.994-5.736） | <0.001* | 1.725（1.108-2.687） | 0.016 |
| Parents/Grandparents/Atavism | 2.627（1.512-4.565） | 0.001* | 1.063（0.516-2.192） | 0.868 |
| Intensity of Physical Activity |  |  |  |  |
| Slight | Ref. |  | Ref. |  |
| Low | 1.332（0.916-1.938） | 0.134 | 1.333（0.838-2.122） | 0.225 |
| Moderate | 2.043（1.486-2.808） | <0.001* | 1.329（0.887-1.991） | 0.168 |
| High | 6.962（4.630-10.470） | <0.001* | 2.590（1.502-4.467） | 0.001 ^a^ |
| Frequency of Physical activities |  |  |  |  |
| 1~3/Month | Ref. |  | Ref. |  |
| 1~2/Week | 1.815（1.348-2.445） | <0.001* | 1.469（0.990-2.180） | 0.056 |
| 3~7/Week | 2.605（1.877-3.615） | <0.001* | 1.978（1.256-3.113） | 0.003 ^a^ |
| Daily Intake（Per Day） |  |  |  |  |
| ≤1 | Ref. |  | Ref. |  |
| ＞1 | 3.013（2.009-4.520） | <0.001* | 2.132（1.321-3.439） | 0.002 ^a^ |
| Sun-sensitivity Skin |  |  |  |  |
| No | Ref. |  | Ref. |  |
| Redness | 1.945（1.448-2.612） | <0.001* | 1.276（0.882-1.846） | 0.196 |
| Burning | 5.240（3.584-7.662） | <0.001* | 2.129（1.310-3.462） | 0.002 ^a^ |
| Blisters | 5.205（2.807-9.652） | <0.001* | 1.882（0.843-4.202） | 0.123 |
| Sleep duration(hours) |  |  |  |  |
| ≥8 | Ref. |  | Ref. |  |
| ＜8 | 2.942（2.278-3.801） | <0.001* | 1.401（1.003-1.957） | 0.048 |
| Dietary structure |  |  |  |  |
| Vegetarian diet | Ref. |  | Ref. |  |
| Red diet | 2.126（1.493-3.027） | <0.001* | 1.313（0.843-2.047） | 0.229 |
| White diet | 0.998（0.680-1.464） | 0.991 | 1.041（0.642-1.687） | 0.872 |
| * significant at P <0.05, ^a^ significant at *P* <0.003 | | | | |


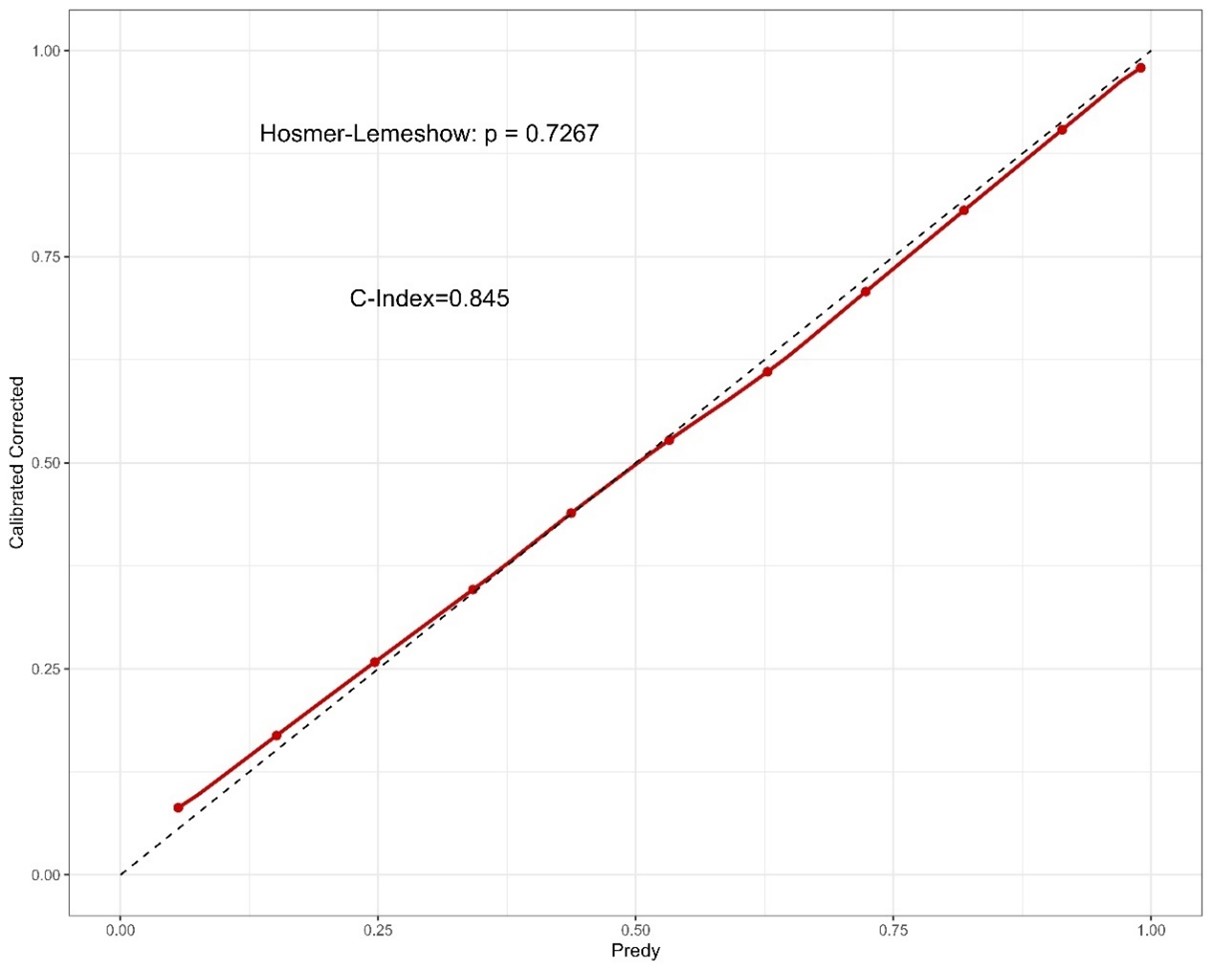


**eFigure 1.** Calibration Curve（Logistic）


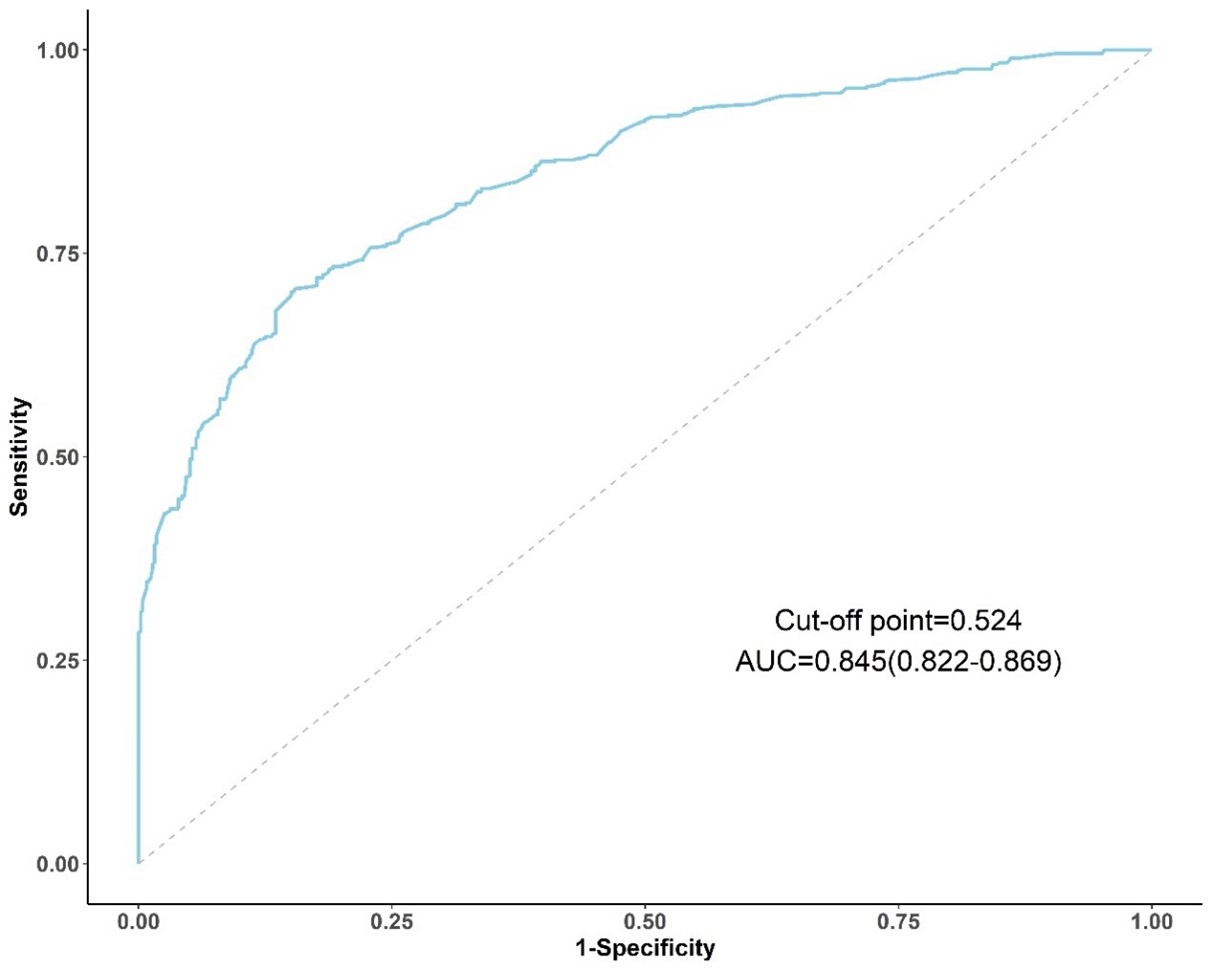


**eFigure 2.** ROC of the risk prediction model for EMs（Logistic）

**eTable 2.** All variables screened by LASSO regression

| Variables | Regression coefficient |
| --- | --- |
| Age | 0 |
| BMI | 0 |
| Age of Menarche | 0 |
| Menstrual Cycle | 0 |
| Duration of Menstruation | 0 |
| Dysmenorrhea Frequency | 0.535 |
| Onset of Dysmenorrhea | 0.211 |
| Degree of Dysmenorrhea | 0 |
| Degree of Dysmenorrhea distress | 0.070 |
| Pelvic Pain of Non-Menstrual Period | 0 |
| Family History of Dysmenorrhea | 0 |
| Family member smoking history | 0 |
| Intensity of Physical Activity | 0.236 |
| Frequency of Physical Activity | 0.163 |
| Daily Intake | 0.305 |
| Sun-sensitivity Skin | 0.198 |
| Sleep Duration | 0.271 |
| Dietary Structure | 0 |
|  |  |


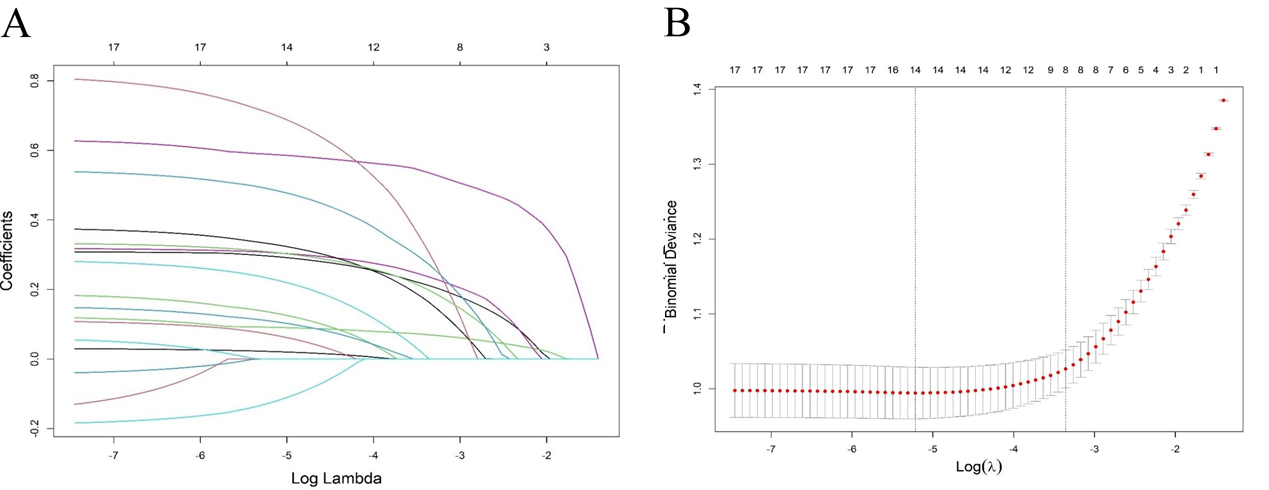


**eFigure 3.** Dysmenorrhea Feature in Patients with Dysmenorrhea Selection by LASSO

(A) To differentiate EMs in patients with dysmenorrhea, LASSO regression was used for variable screening. The results showed that 8 variables were retained when the error was the smallest; that is, the place corresponding to the dotted line on the left. (B) LASSO coefficient profiles of the 18 dysmenorrhea features. A coefficient profile plot was produced against the log(λ) sequence. A vertical line was drawn at the selected optimizing value (λ), which resulted in 8 nonzero coefficients.

**eTable 3.** Univariate and multivariate logistic regression analysis for risk of EMs

|  | Univariate | | Multivariate | |
| --- | --- | --- | --- | --- |
|  | OR（95%CI）  （95%CI）（95%CI） | *P* value | OR（95%CI） | *P* value |
| Dysmenorrhea Frequency |  |  |  |  |
| Never | Ref. |  | Ref. |  |
| Occasionally | 1.734（1.227-2.449） | 0.002 | 0.468（0.246-0.873） | 0.019 |
| Often | 8.414（5.66-12.506） | <0.001* | 1.496（0.709-3.128） | 0.286 |
| Always | 30.411（16.809-55.020） | <0.001* | 4.085（1.620-10.541） | 0.003* |
| Onset of Dysmenorrhea (months) |  |  |  |  |
| <6 | Ref. |  | Ref. |  |
| 6-12 | 1.501（0.939-2.398） | 0.089 | 1.188（0.629-2.239） | 0.594 |
| >12 | 5.986（4.510-7.946） | <0.001* | 3.549（2.226-5.736） | <0.001* |
| Degree of dysmenorrhea distress | 1.370（1.311-1.433） | <0.001* | 1.151（1.060-1.250） | <0.001* |
| Intensity of Physical Activity |  |  |  |  |
| Slight | Ref. |  | Ref. |  |
| Low | 1.332（0.916-1.938） | 0.134 | 1.365（0.864-2.155） | 0.182 |
| Moderate | 2.043（1.486-2.808） | <0.001* | 1.325（0.888-1.978） | 0.167 |
| High | 6.962（4.630-10.47） | <0.001* | 3.015（1.787-5.126） | <0.001* |
| Frequency of Physical activities |  |  |  |  |
| 1~3/Month | Ref. |  | Ref. |  |
| 1~2/Week | 1.815（1.348-2.445） | <0.001* | 1.529（1.039-2.260） | 0.032 |
| 3~7/Week | 2.605（1.877-3.615） | <0.001* | 2.213（1.428-3.449） | <0.001* |
| Daily Intake |  |  |  |  |
| ＞1 | Ref. |  | Ref. |  |
| ≤1 | 3.013（2.009-4.520） | <0.001* | 2.133（1.337-3.467） | 0.002* |
| Sun-sensitivity Skin |  |  |  |  |
| No | Ref. |  | Ref. |  |
| Redness | 1.945（1.448-2.612） | <0.001* | 1.438（1.001-2.071） | 0.050 |
| Burning | 5.240（3.584-7.662） | <0.001* | 2.357（1.469-3.798） | <0.001* |
| Blisters | 5.205（2.807-9.652） | <0.001* | 1.872（0.861-4.122） | 0.115 |
| Sleep duration |  |  |  |  |
| ≥8 | Ref. |  | Ref. |  |
| ＜8 | 2.942（2.278-3.801） | <0.001* | 1.603（1.159-2.216） | 0.004* |
| * significant at *P <0.05* | | | | |


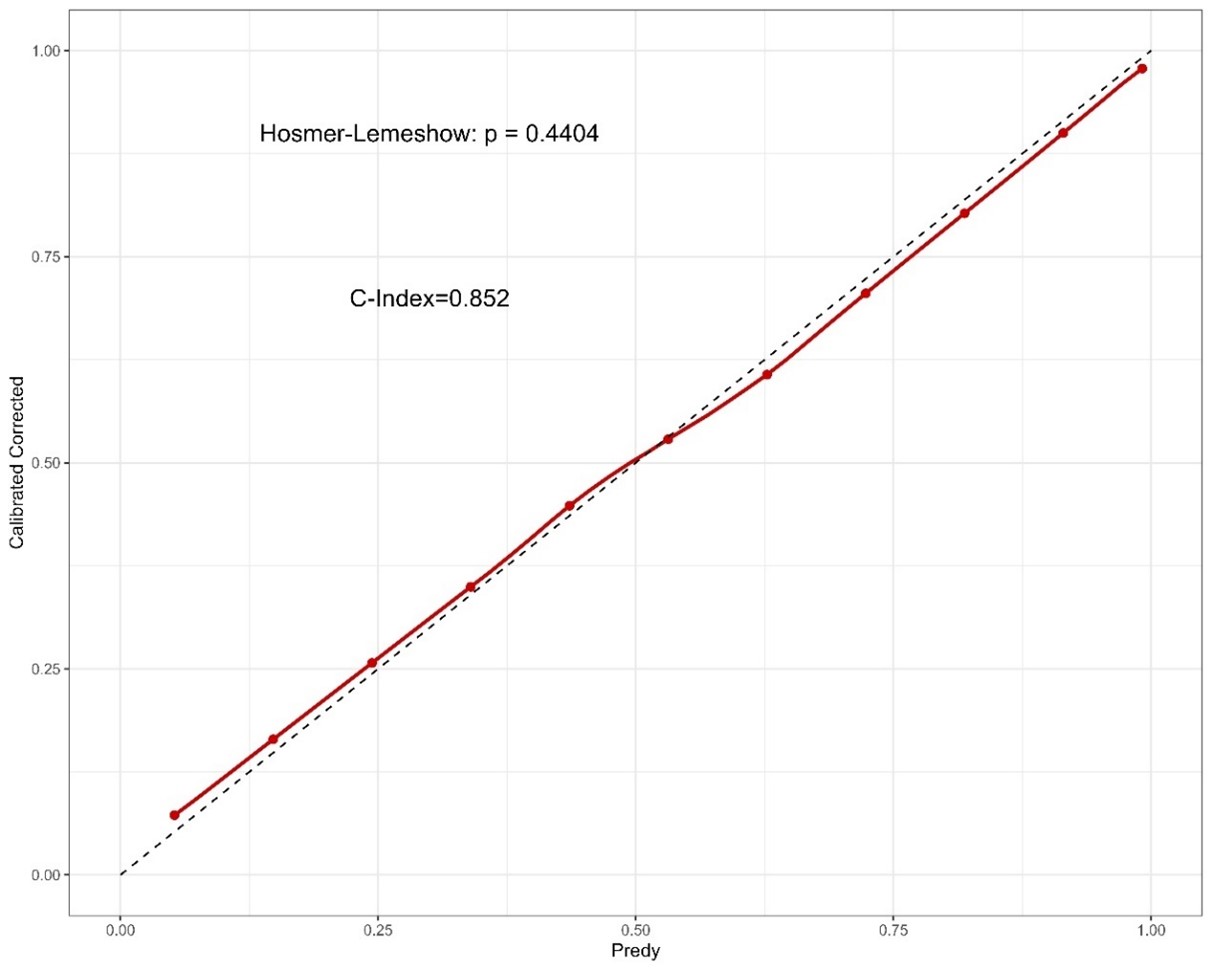


**eFigure 4.** Calibration Curve (LASSO)


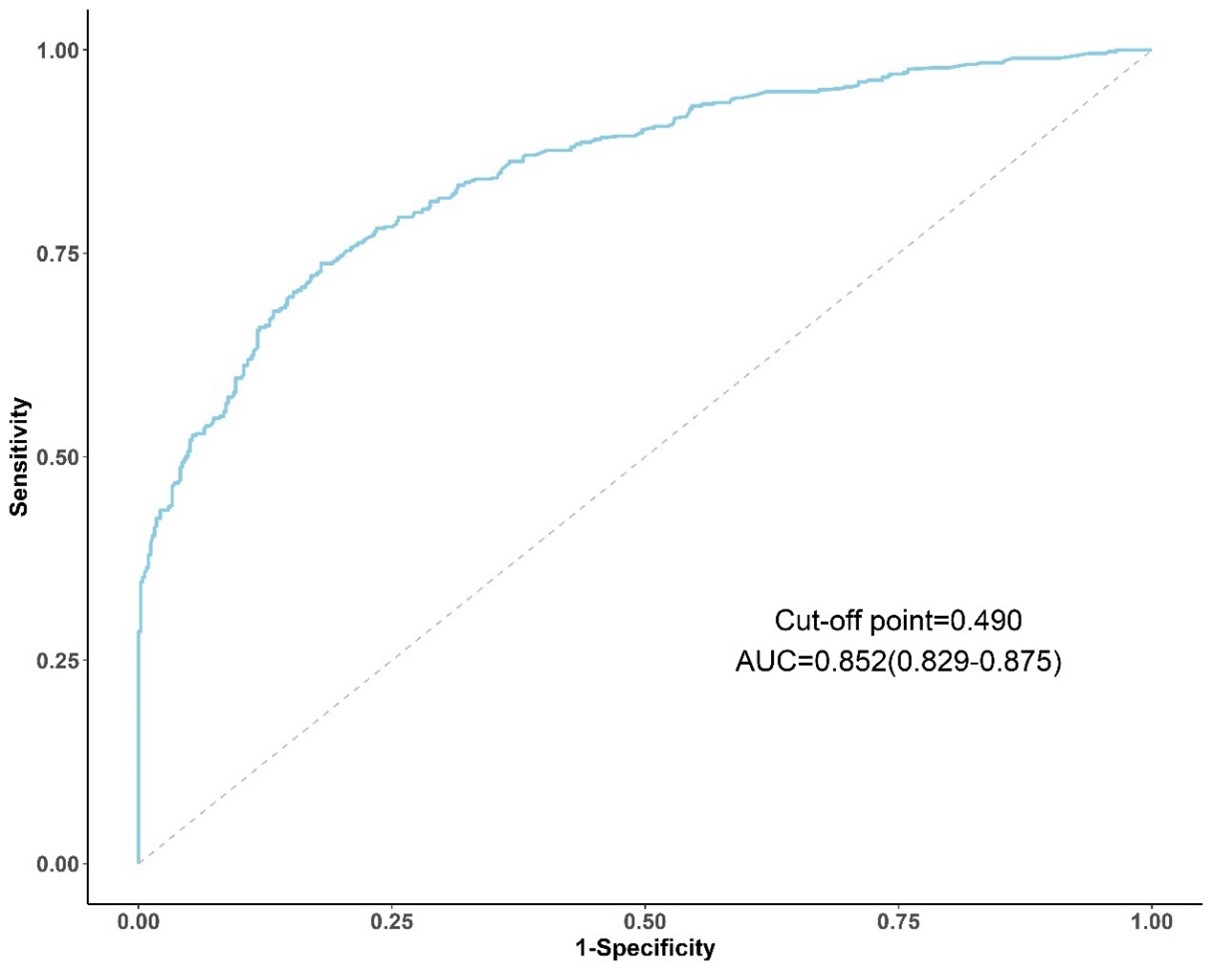


**eFigure 5.** ROC of the risk prediction model for EMs（LASSO）
